# Correlation analysis between the changes in plasma ghrelin level and weight loss after sleeve gastrectomy combined with fundoplication

**DOI:** 10.1186/s12893-024-02468-2

**Published:** 2024-06-05

**Authors:** Xin Li, Aikebaier Aili, Aliyeguli Aipire, Pierdiwasi Maimaitiyusupu, Maimaitiaili Maimaitiming, Kelimu Abudureyimu

**Affiliations:** 1 Department of Minimally Invasive, Hernia and Abdominal Surgery, People’s Hospital of Xinjiang Autonomous Region, Urumqi, Xinjiang Uygur Autonomous Region 830011 China; 2 Graduate School, Xinjiang Medical University, Urumqi, Xinjiang Uygur Autonomous Region 830054 China; 3 Institute of General Surgery and Minimally Invasive Surgery, Xinjiang Uygur Autonomous Region, Urumqi, 830011 China; 4 Clinical Research Center for Gastroesophageal Reflux Disease and Weight Loss and Metabolic Surgery, Xinjiang Uygur Autonomous Region, Urumqi, Xinjiang Uygur Autonomous Region 830011 China

**Keywords:** Obesity, Sleeve gastrectomy, Fundoplication, Ghrelin, Weight loss effect

## Abstract

**Background:**

Laparoscopic sleeve gastrectomy combined with fundoplication (LSGFD) can significantly control body weight and achieve effective anti-reflux effects. The aim of this study is to investigate the correlation between the alteration in Ghrelin levels and weight loss following SGFD, and to compare Ghrelin levels, weight loss and metabolic improvements between SG and SGFD, with the objective of contributing to the existing body of knowledge on SGFD technique in the management of patients with obesity and gastroesophageal reflux disease (GERD).

**Methods:**

A retrospective analysis was conducted on the clinical data of 115 obese patients who underwent bariatric surgery between March 2023 and June 2023 at the Department of Minimally Invasivew Surgery, Hernia and Abdominal Wall Surgery, People’s Hospital of Xinjiang Uygur Autonomous Region. The subjects were divided into two groups based on surgical methods: sleeve gastrectomy group (SG group, 93 cases) and sleeve gastrectomy combined with fundoplication group (SGFD group, 22 cases). Clinical data, such as ghrelin levels before and after the operation, were compared between the two groups, and the correlation between changes in ghrelin levels and weight loss effectiveness after the operation was analyzed. Results: Three months after the operation, there was no significant difference in body mass, BMI, EWL%, fasting blood glucose, triglyceride, cholesterol, and uric acid levels between the SG and SGFD groups (*P* > 0.05). However, the SGFD group exhibited a significant decrease in body weight, BMI, and uric acid levels compared to preoperative levels (*P* < 0.05), while the decrease in ghrelin levels was not statistically significant (*P* > 0.05). Logistic regression analysis indicated that ghrelin levels three months after the operation were influential in postoperative weight loss.

**Conclusion:**

The reduction of plasma Ghrelin level in patients after SGFD is not as obvious as that in patients after SG, but it can make obese patients get the same good weight loss and metabolic improvement as patients after SG. Ghrelin level at the third month after operation is the influencing factor of postoperative weight loss.

## Introduction

Obesity has emerged as a global public health concern in the 21st century [1]. Over the past 50 years, the prevalence of obesity worldwide has steadily increased, reaching pandemic levels [2]. Diet regulation, exercise, and simple medical interventions often fail to yield satisfactory results for obese patients, leading to a growing interest in surgical interventions as effective treatment options [3]. Research suggests that early surgical intervention for obesity, within appropriate indications, can enhance treatment effectiveness [4]. Surgical approaches for obesity treatment include gastric banding, Sleeve gastrectomy (SG), and Roux-en-*γ* gastric bypass (RYGB), among others. SG not only facilitates weight loss but also improves obesity-related comorbidities. By reducing gastric volume by 75–80%, SG is characterized by its low technical difficulty, high safety, and significant short-term and medium-term treatment outcomes [5]. However, gastroesophageal reflux disease (GERD) is a common complication following SG, with an incidence rate of 20%-60% [6]. The presence of Hiatal hernia (HH) prior to surgery further increases the risk of postoperative GERD. Recent studies by the author’s research team have demonstrated that laparoscopic fundoplication with sleeve gastrectomy (LFDSG) can effectively manage body weight and mitigate reflux symptoms [7]. The remission rate of reflux symptoms in patients who underwent SGFD can reach 86.4% one year after operation and 90.9% after an average follow-up of 34 (22–48) months. No medium or long-term complications.

In 1999, the discovery of the gastrointestinal hormone ghrelin, also known as the hunger or growth hormone, greatly advanced our understanding of appetite control mechanisms [8]. Ghrelin, an endogenous brain-gut peptide consisting of 28 amino acid residues, is primarily secreted by X/A-like cells in the stomach’s submucosa. It plays a pivotal role in regulating sugar and lipid metabolism, energy balance, and impact on appetite. SG involves the removal of gastric fundus tissue, resulting in reduced Ghrelin synthesis in the gastric fundus mucosa. This reduction in Ghrelin levels curtails appetite and food intake, thereby facilitating weight loss [9]. However, there is a paucity of research on changes in Ghrelin levels following LFDSG and their correlation with weight loss.

On the basis of the above research [7], the aim of this study is to investigate the correlation between the alteration in Ghrelin levels and weight loss following SGFD, and to compare Ghrelin levels, weight loss and metabolic improvements between SG and LSGFD, with the objective of contributing to the existing body of knowledge on LSGFD technique in the management of patients with obesity and GERD.

## Materials and methods

### Research participants

**Fig. 1:**
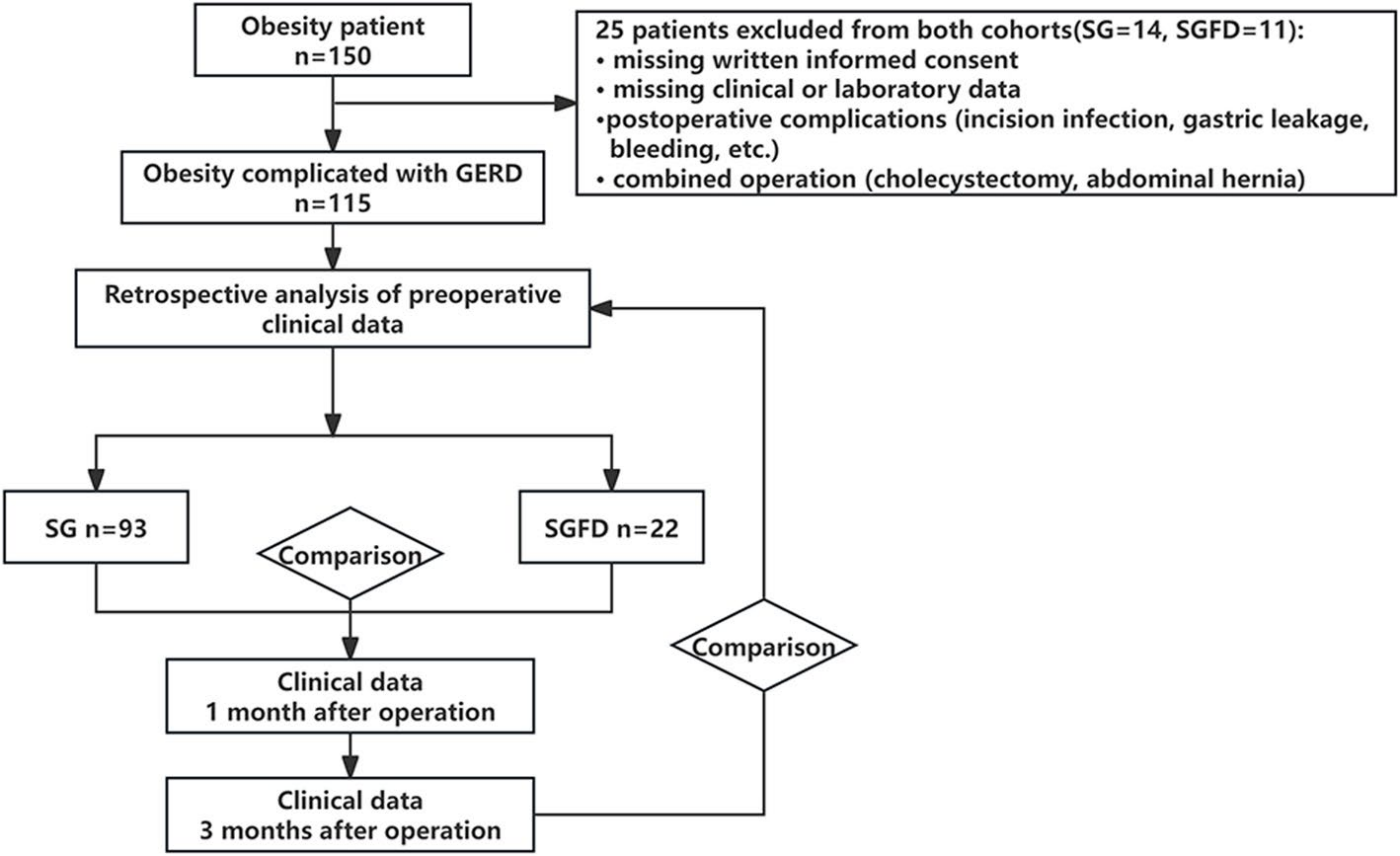
Flow chart for inclusion and exclusion of patients between March 2023 to June 2023 GERD = gastroesophageal reflux disease; SG = sleeve gastrectomy; SGFD = sleeve gastrectomy combined with fundoplication

This retrospective analysis examined the clinical data of 115 individuals with obesity complicated by GERD who underwent bariatric surgery at the Minimally Invasive Surgery, Hernia and Abdominal Surgery department of the People’s Hospital of Xinjiang Uygur Autonomous Region between March 2023 and June 2023. Based on the surgical approach employed, the subjects were divided into two groups: sleeve gastrectomy group (SG group, 93 cases) and sleeve gastrectomy combined with fundoplication group (SGFD group, 22 cases), as depicted in Fig. 1. The study population consisted of 37 males and 78 females, with an average age of (37.37 ± 9.34) years. The mean preoperative body mass was (125.42 ± 26.27) kg, and the average preoperative BMI was (44.37 ± 7.27) kg/m^2^. Comparing the basic clinical characteristics of the two groups, no statistically significant differences were found (*P* > 0.05), indicating their comparability (Table 1).

**Table 1:** Comparison of basic clinical data between 2 groups of patients

Clinical data	SGFD group	SG group	□χ^*2*^*/t*	*P*
Number of cases	22	93		
Age (years, $\overline{x }$ ± s)	38.36 ± 9.62	37.13 ± 9.31	0.556	0.579
Gender [n(%)]			0.219	0.640
Male	8(36.4)	29(31.2)		
Female	14(63.6)	64(68.8)		
Body mass (kg, $\overline{x }$ ±s)	121.76 ± 19.10	126.28 ± 27.71	-0.724	0.471
BMI (kg/m^2^, $\overline{x }$ ±s)	42.75 ± 5.32	44.75 ± 7.64	-1.164	0.247
Hypertension [n(%)]			2.783	0.095
Yes	6(27.3)	12(12.9)		
No	16(72.7)	81(87.1)		
Type 2 diabetes [n(%)]			2.280	0.131
Yes	6(27.3)	13(14.0)		
No	16(72.7)	80(86.0)		

*BMI* Body mass index, body mass divided by the square of height

### Inclusion criteria and exclusion criteria

The inclusion criteria for this study were as follows: (1) Age between 18 and 65 years, with a BMI ≥ 30 kg/m^2^ and a confirmed diagnosis of obesity complicated by GERD; (2) Accurate and comprehensive recording of clinical data such as body weight, height, and BMI, as well as precise documentation of plasma ghrelin levels, blood sugar, triglyceride, cholesterol, and uric acid levels before and after the operation; (3) Ineffectiveness of conservative treatment and a need for surgical intervention, meeting the indications for weight loss metabolic surgery; (4) Signed informed consent form.

Indications of metabolic and bariatric surgery: Metabolic and bariatric surgery (MBS) is recommended for individuals with a body mass index (BMI) > 35 kg/m^2^, regardless of presence, absence, or severity of co-morbidities. MBS should be considered for individuals with metabolic disease and BMI of 30–34.9 kg/m^2^. Individuals with BMI > 27.5 kg/m^2^ should be offered MBS in the Asian population [10].

Diagnostic criteria of GERD: (1) Typical symptoms such as acid reflux, heartburn and retrosternal pain; (2) Proton pump inhibitor test is positive; (3) Endoscopic examination revealed reflux esophagitis; (4) 24 h esophageal pH monitoring indicates reflux; (5) GERD Q scale score ≥ 8; (6) high-resolution manometry (HRM) can be used to determine whether there is esophageal hiatal hernia, which is mainly manifested by double pressure bands in the lower esophagus, the reversal point of respiratory pressure moving down, and the lower esophageal sphincter pressure (LESP) decreasing below the normal value.

The exclusion criteria were as follows: (1) Presence of severe comorbidities that render the patient unable to tolerate surgical treatment or contraindicate surgery; (2) Absence of important research information related to patients’ weight loss metabolism including written informed consent, clinical or laboratory data; (3) Refusal to participate in the study involving sleeve gastrectomy combined with fundoplication; (4) Refusal to undergo relevant examinations. Prior to participating in the study, all participants provided informed consent for the use of their clinical data for scientific purposes.

This study adheres to the principles outlined in the Helsinki Declaration and has received approval from the Medical Ethics Committee of the People’s Hospital of Xinjiang Uygur Autonomous Region (batch number: KY2020041007).

### Research method

Upon admission, the patients’ height and weight were measured both pre- and post-operation, enabling the calculation of their BMI. Relevant clinical data, such as age, sex, complications, and surgical methods, were collected from the hospital’s electronic medical record system. Fasting blood samples were obtained in the morning to assess levels of serum blood sugar, triglyceride, cholesterol, and uric acid. The percentage of excess weight loss (%EWL) was calculated at one month and three months following the operation using the formula: %EWL = (original body mass—decreased body mass)/(original body mass—target body mass) × 100%. The target body mass was set as the body mass when BMI equals 25 kg/m^2^. Based on %EWL, the weight loss outcomes were categorized as excellent (≥ 75%), good (50%- < 75%), effective (25%- < 50%), or ineffective (< 25%).

### Surgical methods

The basis for choosing the operation method is as follows: Inform patients in detail about the results of preoperative examination and the possible related risks of SGFD. The operation mode is mainly determined according to the severity of preoperative reflux symptoms, Gerd score, the degree of LESP reduction and the patient’s wishes. The surgical methods include SG and SGFD.

**Fig. 2:**
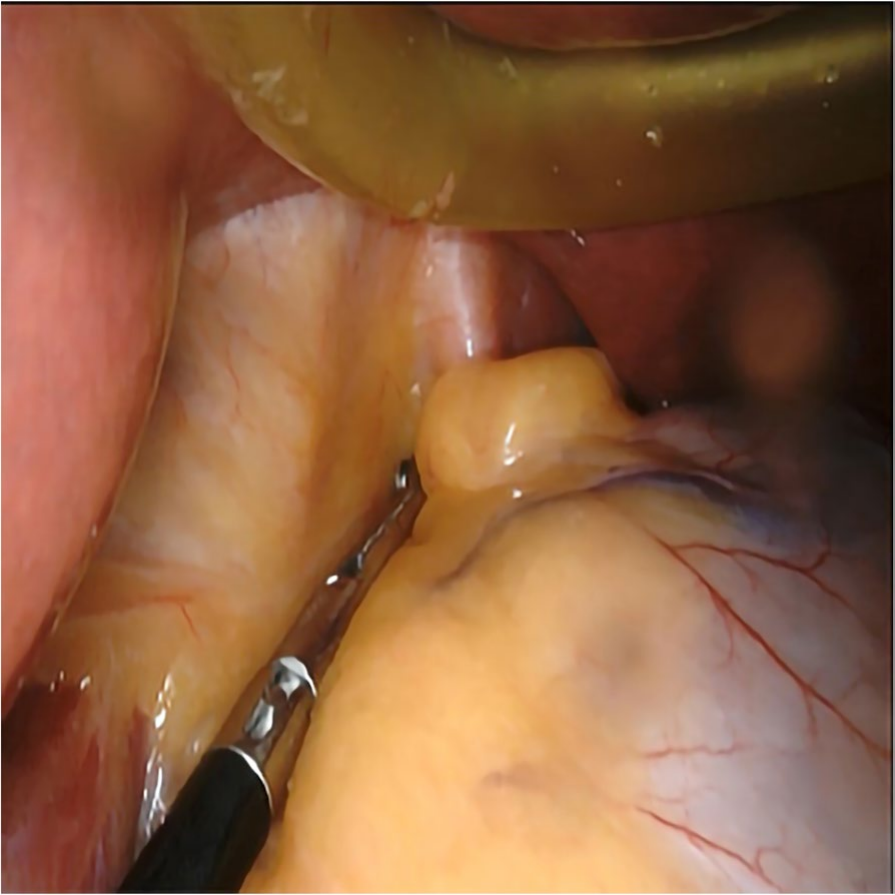
Exploration revealed esophageal hiatal hernia

**Fig. 3:**
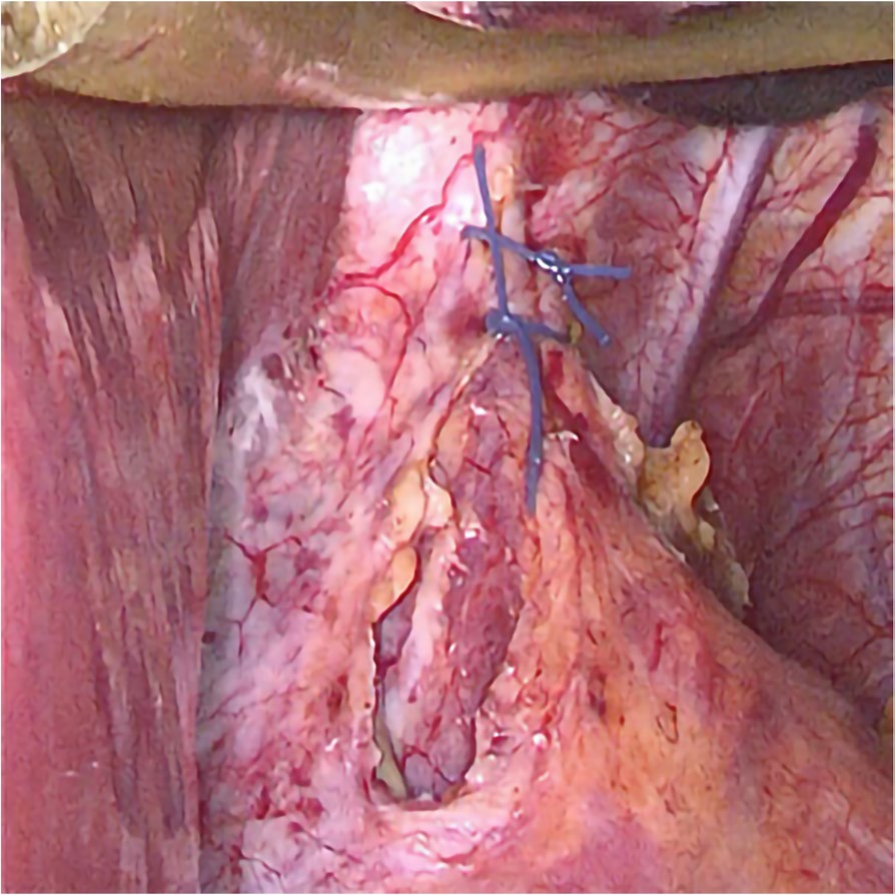
Repair of hiatal hernia in front of the esophagus

Anesthesia and surgical methods in the SG Group: (1) A combination of intravenous and inhalational general anesthesia was administered to the patient, who was placed in a supine position. The patient was disinfected and covered with a cloth. (2) The abdomen was explored after establishing pneumoperitoneum. If a hiatal hernia is found, it needs to be repaired, as shown in Figs. 2 and 3. The gastrocolon ligament was severed using an ultrasonic scalpel to fully release the gastric fundus and expose the left crura of the diaphragm. (3) For the cutting guidance, a 36 Fr gastric correction tube was orally inserted. (4) Utilizing a gastric correction tube as a guide, an incision was made on the greater curvature of the stomach at a distance of 3 cm from the pylorus, using a straight-line cutting closure device to perform a sleeve-shaped resection. (5) The gastric stapling was continuously sutured to reinforce the edge. The greater omentum was reset and fixed, followed by the removal of the specimen and placement of a drainage tube. Finally, the operation was concluded.

**Fig. 4:**
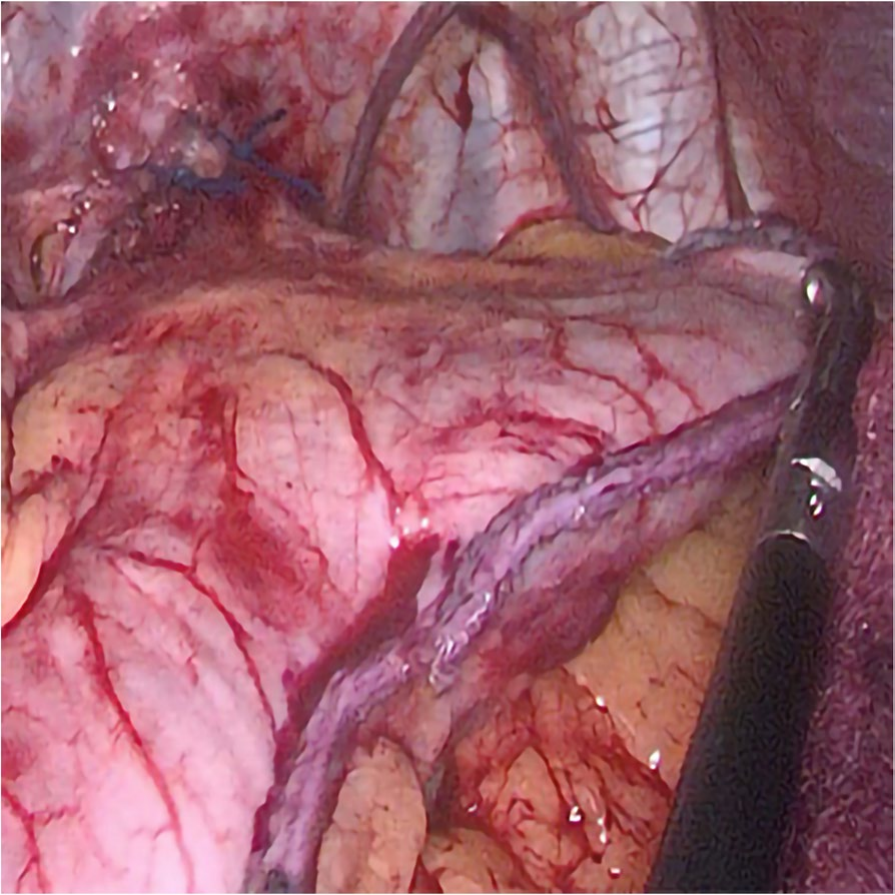
Keep a suitable part of the fundus for folding

**Fig. 5:**
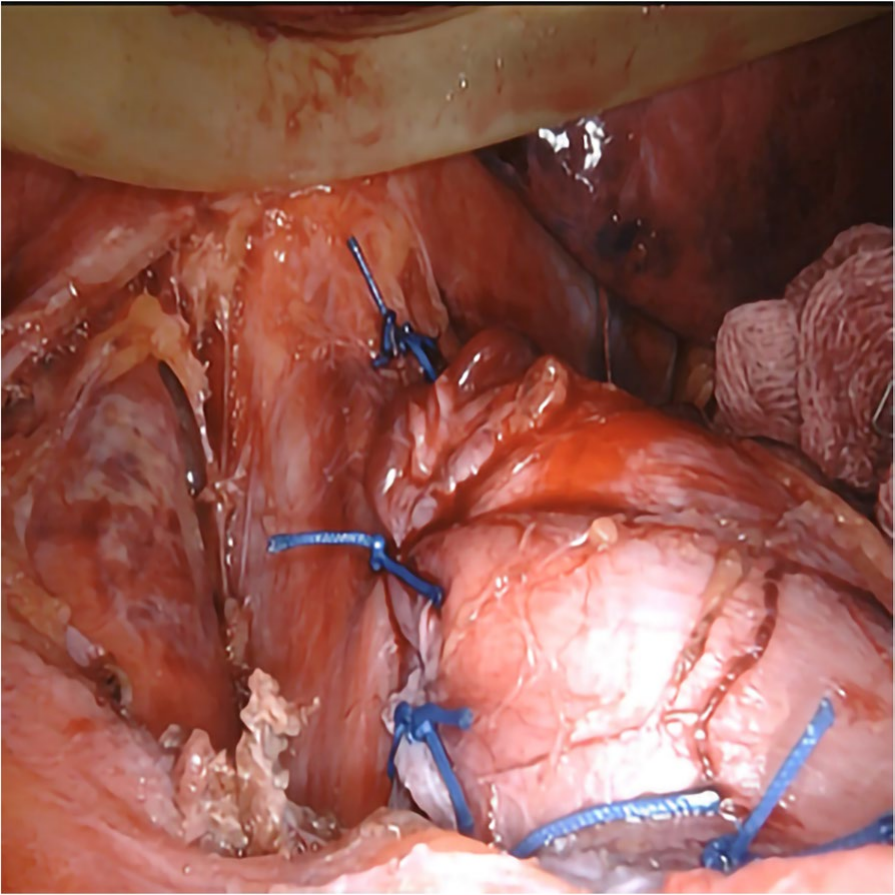
180-degree folding of the gastric fundus (Dor folding)

Anesthesia and surgical methods in the SGFD group: (1) The surgical position, anesthesia, disinfection, and towel placement were carried out in the same manner as in the SG group. The pneumoperitoneum was established, the gastrocolon ligament was disconnected, and a gastric correction tube was inserted. (2) Starting 3 cm from the pylorus, a section of the larger curve of the stomach was excised, leaving a width of approximately 3–4 cm and a length of 6–8 cm, or enough to allow for sufficient folding of the gastric fundus. The leaving gastric fundus is similar to a fan shape, and the length of the hypotenuse near the cardia is 3–4 cm, and the length of the hypotenuse near the greater curvature of the stomach is 6–8 cm, as shown in Fig. 4. The gastric stapling edge was reinforced with continuous sutures, and the greater omentum was reset and immobilized. (4) The preserved gastric fundus was folded and wrapped around the esophagus, with the folding technique determined based on preoperative examination results. (5) The folded flap was secured to the diaphragm, as depicted in Fig. 5. (6) Following specimen removal, a drainage tube was placed, marking the completion of the operation. During the operation, excessive free posterior wall of gastric fundus and exposed His angle should be avoided, and the blood supply and venous return of gastric fundus artery should be protected as much as possible to prevent ischemic gastric leakage. We first excised the larger curve of the stomach, and then reinforced the gastric stapling. During the reinforcement of gastric stapling, the blood supply of the gastric fundus can be fully observed. If the fundus of the stomach has poor blood supply such as congestion and ischemia, for the sake of patient safety, the preserved fundus of the stomach should be removed and the fundoplication should be abandoned.

### Detection of plasma Ghrelin

The levels of plasma ghrelin were measured preoperatively, as well as at 3 days, 1 month, and 3 months postoperatively. The following detection procedure was used: In the morning, fasting venous blood samples were collected from the patients, and the plasma was separated by centrifugation and stored in plastic storage tubes. The samples were then kept at -80^*◦*^*C* until further analysis. The Ghrelin content in the plasma was quantified using an enzyme-linked immunosorbent assay (ELISA) method with the Ghrelin human kit (CEA 991Hu, Wuhan USCN Business Co., Ltd., Wuhan, CHN).

### Research contents

The study aimed to compare ghrelin levels, weight loss effects, and metabolic improvements between the two groups. Differences in Ghrelin levels, BMI, %EWL (percentage of excess weight loss), blood sugar, triglyceride, cholesterol, and uric acid levels were analyzed between the two groups at 1 month and 3 months postoperatively. Additionally, the changes in Ghrelin levels, weight loss effects, and metabolic improvements in both groups were examined. Based on the %EWL three months after the operation, the weight loss effects were categorized as either good (≥ 50%) or average (< 50%). The relationship between Ghrelin levels and a significant weight loss effect three months after surgery, as well as the factors influencing this positive outcome, were analyzed using logistic regression.

### Statistical method

Statistical analysis was performed using SPSS 22.0 software. Measurement data were presented as mean ± standard deviation (s), and a t-test was conducted to compare the two groups. Counting data were expressed as percentages (%), and a chi-square test was employed to compare the two groups. A *p*-value < 0.05 was considered statistically significant. Logistic regression analysis was used to examine the relationship between Ghrelin levels and successful weight loss three months after surgery, as well as the factors influencing this positive outcome.

## Results

### Comparison of clinical data between two groups

There were no significant differences in plasma Ghrelin levels between the two groups before the operation, at 3 days, 1 month, and 3 months post-operation (*P* > 0.05). Similarly, no significant differences were observed in fasting blood glucose, triglyceride, cholesterol, and uric acid levels before the operation (*P* > 0.05). One month after surgery, there were no significant differences between the two groups in terms of body mass, BMI, %EWL, cholesterol, and uric acid levels. Additionally, there were no significant differences between the two groups in terms of body mass, BMI, %EWL, fasting blood glucose, triglycerides, cholesterol, and uric acid levels at 3 months post-surgery (*P* > 0.05). However, one month after surgery, the SGFD group exhibited significantly lower fasting blood glucose levels (4.44 ± 0.50 vs. 4.93 ± 1.12, *P* = 0.047) and triglyceride levels (1.11 ± 0.36 vs. 1.64 ± 0.63, *P* < 0.001) compared to the SG group, indicating a statistically significant difference (*P* < 0.05) (Table 2).

**Table 2:** Comparison of 2 groups of clinical data

Clinical data	SGFD group	SG group	χ^*2*^*/t*	*P*
Number of cases	22	93		
Preoperative Ghrelin/*10^2^ pg·ml^−1^	12.35 ± 2.33	16.19 ± 1.49	-1.178	0.241
Ghrelin 3 days after operation/*10^2^ pg·ml^−1^	11.08 ± 2.33	10.61 ± 1.19	0.174	0.862
Preoperative fasting blood glucose/mmol·L^−1^	4.76 ± 0.95	5.12 ± 2.05	-0.794	0.429
Preoperative triglyceride/mmol·L^−1^	1.26 ± 0.80	2.22 ± 0.27	-1.719	0.088
Preoperative cholesterol/mmol·L^−1^	4.30 ± 0.68	4.86 ± 1.57	-1.631	0.106
Preoperative uric acid/μmol·L^−1^	423.94 ± 126.25	431.92 ± 132.82	-0.256	0.799
Body mass 1 month after operation(kg, $\overline{x }$±s)	106.12 ± 16.34	110.87 ± 23.67	-0.890	0.376
BMI 1 month after operation(kg/m^2^, $\overline{x }$±s)	37.29 ± 4.80	39.34 ± 6.80	-1.335	0.185
%EWL 1 month after operation $\overline{x }$±s)	32.15 ± 9.28	30.59 ± 14.85	0.470	0.639
Ghrelin 1 month after operation/*10^2^ pg·ml^−1^	12.94 ± 1.78	11.13 ± 1.03	0.796	0.428
Fasting blood glucose 1 month after operation/mmol·L^−1^	4.44 ± 0.50	4.93 ± 1.12	-2.008	0.047
Triglyceride 1 month after operation/mmol·L^−1^	1.11 ± 0.36	1.64 ± 0.63	-3.762	< 0.001
Cholesterol 1 month after operation/mmol·L^−1^	3.85 ± 0.65	4.19 ± 0.80	-1.828	0.070
Uric acid 1 month after operation/μmol·L^−1^	376.51 ± 174.36	443.33 ± 168.48	-1.662	0.099
Body mass 3 month after operation(kg, $\overline{x }$±s)	91.12 ± 14.12	95.97 ± 20.13	-1.068	0.288
BMI 3 month after operation(kg/m^2^, $\overline{x }$±s)	32.07 ± 4.49	34.07 ± 5.92	-1.487	0.140
%EWL 3 month after operation $\overline{x }$±s)	63.35 ± 15.41	60.03 ± 25.41	0.588	0.558
Ghrelin 3 month after operation/*10^2^ pg·ml^−1^	11.37 ± 2.09	10.56 ± 1.00	0.356	0.722
Fasting blood glucose 3 month after operation/mmol·L^−1^	4.56 ± 0.52	4.63 ± 0.69	-0.455	0.650
Triglyceride 3 month after operation/mmol·L^−1^	1.28 ± 0.33	1.50 ± 0.54	-1.802	0.074
Cholesterol 3 month after operation/mmol·L^−1^	4.13 ± 0.90	4.58 ± 1.02	-1.887	0.062
Uric acid 3 month after operation/μmol·L^−1^	352.02 ± 62.81	389.65 ± 126.44	-1.354	0.178

### Changes in Ghrelin level, body mass and metabolic indexes after SGFD

A comparison was made between the SGFD and SG groups regarding the changes in Ghrelin levels, body mass, BMI, fasting blood glucose, and other metabolic indexes before and three months after the operation. After three months, a significant decrease was observed in the body mass, BMI, and uric acid levels of the SGFD group compared to before the operation. This difference was found to be statistically significant (*P* < 0.05). However, no significant changes were observed in the Ghrelin level, blood sugar, and triglyceride levels in the SGFD group compared to before the operation (*P* > 0.05) (Table 3). In contrast, the SG group displayed significant reductions in body mass, BMI, Ghrelin level, blood sugar, triglyceride, and uric acid levels three months after the operation compared to before the procedure. These differences were statistically significant (*P* < 0.05) (Table 4).

**Table 3:** Comparison of clinical data between SGFD group patients before and 3 months after surgery

Clinical data	Preoperative	3 month after operation	□χ^*2*^*/t*	*P*
Body mass (kg, $\overline{x }$±s)	121.76 ± 19.10	91.12 ± 14.12	19.317	< 0.001
BMI (kg/m^2^, $\overline{x }$±s)	42.75 ± 5.32	32.07 ± 4.49	26.657	< 0.001
Ghrelin/*10^2^ pg·ml^−1^	12.35 ± 2.33	11.37 ± 2.09	0.341	0.736
Fasting blood glucose/mmol·L^−1^	4.76 ± 0.95	4.56 ± 0.52	0.910	0.373
Triglyceride/mmol·L^−1^	1.26 ± 0.80	1.28 ± 0.33	-0.108	0.915
Cholesterol/mmol·L^−1^	4.30 ± 0.68	4.13 ± 0.90	0.696	0.494
Uric acid/μmol·L^−1^	423.94 ± 126.25	352.02 ± 62.81	2.547	0.019

**Table 4:** Comparison of clinical data between SG group patients before and 3 months after surgery

Clinical data	Preoperative	3 month after operation	□χ^*2*^*/t*	*P*
Body mass (kg, $\overline{x }$±s)	125.91 ± 25.44	95.97 ± 20.13	9.420	< 0.001
BMI (kg/m^2^, $\overline{x }$±s)	44.75 ± 7.64	34.07 ± 5.92	36.610	< 0.001
Ghrelin/*10^2^ pg·ml^−1^	16.19 ± 1.49	10.56 ± 1.00	2.962	0.004
Fasting blood glucose/mmol·L^−1^	5.10 ± 2.04	4.63 ± 0.69	2.121	0.037
Triglyceride/mmol·L^−1^	2.22 ± 0.27	1.50 ± 0.54	2.696	0.008
Cholesterol/mmol·L^−1^	4.86 ± 1.57	4.58 ± 1.02	1.541	0.127
Uric acid/μmol·L^−1^	431.92 ± 132.83	389.65 ± 126.44	2.195	0.031

### Logistic regression analysis of the relationship between plasma Ghrelin level and weight loss effect

Based on the %EWL three months after the operation, the patients in both groups were categorized into two groups: those with a good weight loss effect (≥ 50%) and those with an average effect (< 50%). Logistic regression analysis was utilized to examine the factors influencing the weight loss effect. The following variables were considered: age (continuous variable), gender (male = 1, female = 0), BMI (continuous variable), body mass (continuous variable), hypertension (yes = 1, no = 0), type 2 diabetes mellitus (yes = 1, no = 0), operation method (SGFD operation = 1, SG operation = 0), preoperative Ghrelin level (continuous variable), and postoperative Ghrelin level after 3 months (continuous variable). The dependent variable in the logistic regression analysis was the grouping variable (good effect = 1, average effect = 0). The results indicated that age, BMI, body mass, type 2 diabetes mellitus, and Ghrelin level 3 months after the operation were significant factors influencing postoperative weight loss (*P* < 0.05) (Table 5).

**Table 5:** Logistic regression analysis of relevant factors affecting weight loss effectiveness

Factor	*β*	*SE*	Wald *χ*^*2*^	*P*	*OR*	95%CI
Age	-0.045	0.022	4.018	0.045	0.956	0.916 ~ 0.999
Gender	0.477	0.469	1.035	0.309	1.6111	0.643 ~ 4.037
BMI	-0.171	0.040	18.590	< 0.001	0.843	0.780 ~ 0.911
Body mass	-0.021	0.008	6.280	0.012	0.979	0.963 ~ 0.995
Hypertension	0.351	0.610	0.331	0.565	1.420	0.430 ~ 4.691
Type 2 diabetes mellitus	-1.318	0.520	6.436	0.011	0.268	0.097 ~ 0.741
Operation method	0.034	0.532	0.004	0.949	1.035	0.365 ~ 2.933
Preoperative ghrelin level	0.009	0.016	0.312	0.576	1.009	0.977 ~ 1.042
Ghrelin level 3 months after operation	-0.054	0.022	6.213	0.013	0.947	0.907 ~ 0.988

## Discussion

### Relationship between ghrelin and obesity and metabolism

Obesity is a chronic metabolic disorder characterized by excessive accumulation of body fat and overweight. It has multiple causes, including genetic factors, environmental factors, and endocrine abnormalities. Gastrointestinal hormones within the endocrine system play a crucial role in signaling the brain about the arrival of nutrients through the central brain-intestinal axis, thereby exerting significant regulatory effects on appetite. The stomach, as an essential component of the digestive system, not only functions to mechanically break down and grind food, but also secretes gastric acid, pepsin, and various gastrointestinal hormones such as gastrin, motilin, and ghrelin. These hormones play a critical role in regulating gastrointestinal function and appetite [11]. Ghrelin, an endogenous brain-gut peptide, is primarily secreted by X/A-like cells in the oxyntic gland, a submucosal layer of the stomach. It serves as an important regulator of glucose and lipid metabolism, as well as energy balance [8]. Ghrelin, an endogenous ligand of the growth hormone secretagogue receptor (GHSR), not only stimulates the release of growth hormone (GH) from the pituitary gland upon binding with GHSR [12], but also facilitates communication between the central nervous system and peripheral organs and tissues involved in metabolism. This hormone is vital in promoting food intake, regulating lipid metabolism, and maintaining energy balance [13]. In a state of positive energy balance, the production of ghrelin in stomach tissue decreases, leading to a reduction in food intake to maintain energy homeostasis. Consequently, obesity is associated with decreased production of ghrelin in stomach tissue [14]. Conversely, in a state of negative energy balance, the production of ghrelin in stomach tissue increases, stimulating food intake to maintain energy homeostasis. GHSR is primarily found in the arcuate nucleus of the hypothalamus. Upon binding with ghrelin, it activates neuropeptide Y (NPY) and agouti-related peptide (AgRP) neurons, which stimulate appetite, causing hunger and subsequent feeding behavior [15]. GHSR plays a crucial role in regulating appetite and energy balance. Although diet control can increase Ghrelin levels, it ultimately leads to increased food intake, making long-term weight loss difficult to achieve [16]. It has been observed that obese patients exhibit a weakened ability to inhibit Ghrelin secretion after meals, along with decreased secretion of appetite-inhibiting hormones such as Peptide YY (PYY) and Glucagon-like Peptide-1 (GLP-1), thereby disrupting energy homeostasis and contributing to progressive obesity [17]. Obesity will lead to impaired secretion and regulation of ghrelin in stomach tissue, which will lead to ghrelin resistance, and the above functions can be recovered by losing weight [18]. According to the above research and analysis, the resistance of ghrelin is a mechanism aimed at protecting the higher body weight set point established during food supply, so as to store energy to the maximum during food shortage. Ghrelin resistance caused by obesity is mainly related to the increase of affinity between immunoglobulin and Ghrelin in blood caused by high-fat diet, thus protecting it from degradation [19], the decrease of NPY/AgRP responsiveness to ghrelin axis signal and nerve signal conduction disorder [20].

This study also reported higher Ghrelin levels in both groups before surgery compared to after surgery, which may contribute to obesity among obese patients. Furthermore, ghrelin is closely associated with glucose and lipid metabolism. It has been found that the interaction between ghrelin and the GHSR-1a in the pancreas reduces insulin secretion and promotes glucagon secretion, consequently raising blood sugar levels [21]. Animal experiments have confirmed that ghrelin, upon binding with GHSR-1a in the dorsal vagal complex, leads to a significant increase in serum triglyceride and total cholesterol levels, as well as the accumulation of liver lipids, thus influencing lipid metabolism [22]. The author observed that preoperative blood sugar, triglyceride, and cholesterol levels were higher in both groups compared to postoperative levels, potentially indicating a connection to elevated plasma Ghrelin levels before surgery. Therefore, ghrelin modulates appetite, feeding behavior, glucose, and lipid metabolism, ultimately participating in the development and progression of obesity.

### Relationship between Ghrelin changes and weight loss and metabolic improvement after SGFD

Weight loss surgery has emerged as an effective approach for obesity treatment, enabling weight reduction, improved metabolism, and alleviation of obesity-related complications. The success of weight loss surgery in achieving sustained weight loss and maintenance primarily stems from the decrease in postoperative hunger and satiety, resulting in reduced calorie intake [23]. Hunger and satiety are regulated through the interaction of central and peripheral nerves and endocrine signals, including changes in gastrointestinal hormone secretion [24]. Gastrointestinal hormones transmit nutrient signals to the central nervous system via the brain-intestine axis, thereby exerting a potent appetite-regulating effect. Surgical procedures like SG disrupt the integrity of the gastric fundus, leading to a noticeable reduction in Ghrelin levels, loss of appetite, and decreased food intake, thus accomplishing weight loss objectives and providing strong evidence for Ghrelin production in the gastric fundus [25]. A study has demonstrated that the decline in Ghrelin levels following surgery is associated with changes in postoperative eating habits, including reduced appetite for high-sugar and high-fat foods [26]. The reduction in ghrelin after metabolic surgery for weight loss can potentially play a pivotal role in reducing body mass and improving obesity-related conditions [27]. Various surgical methods yield different outcomes in terms of Ghrelin changes, possibly attributed to distinct mechanisms associated with weight loss and metabolic improvement. Research suggests that among obesity surgeries, SG leads to a greater decrease in ghrelin levels compared to other surgical approaches [28]. This study revealed a significant reduction in Ghrelin levels in the SG group post-surgery, with a noteworthy statistical difference. Logistic regression analysis further confirmed the association between ghrelin levels at the third month after surgery and the effectiveness of subsequent weight loss, indicating that lower Ghrelin levels correlated with improved postoperative weight loss outcomes. However, divergent results have been reported by another cohort study [29]. Following SG, fasting Ghrelin levels gradually increased and eventually surpassed preoperative levels. However, the elevation in ghrelin did not impact postoperative weight loss. Hence, some studies propose that incomplete gastrectomy may be an additional factor contributing to the rise in Ghrelin after SG [30]. Furthermore, another investigation discovered a robust positive correlation between plasma Ghrelin levels and the total number of Ghrelin-producing cells in the gastric fundus. This suggests that complete resection of the gastric fundus could effectively reduce Ghrelin levels [31].

After sleeve gastrectomy in SGFD surgery, a part of the gastric fundus with ghrelin—producing cells was retained to wrap around the lower esophagus. At present, there are different results about the change of ghrelin concentration after gastric plication. One study observed that the concentration of ghrelin increased significantly after gastric banding operation (*P* = 0.01) and Nissen fundoplication (*P* = 0.02), but it decreased significantly after RYGB operation (*P* < 0.001), which may be related to the complete preservation of the fundoplication in Nissen [32]. However, another study found that BMI and ghrelin decreased in the first week and the fourth week after Nissen fundoplication, and there was a strong correlation between ghrelin value and BMI change in the fourth week after operation, probably because fundoplication would damage the secretion function of ghrelin in the gastric fundus [33].

There are few studies on the changes of ghrelin concentration after SGFD. In this study, the author observed a non-significant decrease in Ghrelin levels three months after sleeve gastrectomy with fundus preservation, potentially due to the retention of a portion of the gastric fundus. In addition, the reduction of plasma ghrelin level in SGFD patients is not as obvious as that in SG patients. The reasons for the decrease of ghrelin concentration after SGFD may include the following three points: (1) During SGFD, most of the gastric fundus was removed, and only a small part of the gastric fundus was preserved. (2) After SGFD, the cells that produce ghrelin in the fundus of stomach can’t contact with food and nutrients, which impairs the secretion function of ghrelin in the fundus of stomach. (3) With the weight loss and the restoration of energy balance, the resistance of ghrelin gradually returned to normal. Therefore, it should be clearly emphasized that the changes in the level of gut hormone after all metabolic and bariatric surgeries are only temporary and usually return almost to preoperative levels within 1–2 years due to adaptive changes in gut epithelium.

After three months, there was no significant difference in the weight loss effect between the SGFD and SG groups, both achieving the criteria for substantial weight loss. Logistic regression analysis confirmed that the surgical method did not influence the weight loss effect three months postoperatively. Therefore, the weight loss mechanism following SGFD may be associated with a decrease in stomach capacity and the alteration of eating habits, as food passes through the gastroesophageal junction at a slower pace after gastric folding. Additionally, the research team’s previous investigations have shown remarkable weight loss effects of gastric fundus folding anti-reflux surgery [7]. We found an interesting phenomenon in the changes of blood sugar and triglyceride after SGFD. One month after surgery, the SGFD group significantly lower fasting blood glucose levels and triglyceride levels compared to the SG group. After SGFD, the LESP increased and the speed of food passing through the gastroe-sophageal junction slowed down. It has been reported that more than 50% of patients began to have dysphagia one week after operation, so they must develop the eating habit of”eating less and eating more, chewing slowly” after operation [34]. One study confirmed that fundoplication can lead to significant and lasting weight loss [35]. Dysphagia, changes in eating habits and weight loss may be the reasons for the obvious difference in blood sugar and triglyceride levels between the two groups one month after operation. However, the intra-group comparison found that the improvement in blood sugar and triglyceride levels in the SGFD group was less significant than in the SG group, three months after surgery. Further follow-up and observation are required to determine whether this discrepancy is related to the difference in Ghrelin levels. Three months after surgery, the two groups had no notable differences in blood glucose and lipid levels. There was no significant difference in blood glucose, triglyceride and cholesterol levels between the two groups before and 3 months after operation, which indicated that the SGFD group could achieve the same metabolic improvement effect as the SG group after operation.

The limitation of this study was that it is a retrospective single-center study, which is prone to bias such as selection and recall bias. In addition, another limitation of this study was that there is a significant difference in sample size between the two groups, which may have a certain impact on the interpretation of the research results. The sample size of the SGFD group is too small, which may lead to insufficient research efficiency, unable to reflect the real differences, and may lead to selective bias, which may not represent the population well and affect the extrapolation of the results. When interpreting the results, it is necessary to consider the possible impact of sample size differences, and even statistically significant results may be affected by accidental factors. In future research, we should consider using larger samples of the SGFD group to further improve the reliability and extrapolation of research results. 3 months follow-up is a short period for evaluation. The short follow-up time is also one of the limitations of this study. Nevertheless, the limited follow-up duration necessitates further long-term research to examine the relationship between Ghrelin levels, improvement in glucose and lipid metabolism following SGFD, and the underlying molecular mechanisms. Regarding the molecular mechanism of the interaction between ghrelin level and metabolic indicators, the team is currently carrying out relevant basic experimental research. It is crucial to conduct multidisciplinary and multicenter clinical investigations promptly to establish high-quality evidence for clinical diagnosis and treatment. This will allow for the provision of robust and reliable evidence-based medical support for SGFD surgery in managing obese patients with GERD, ultimately elevating the standard of diagnosis and treatment.

## Conclusion

The reduction of plasma ghrelin level in patients after SGFD is not as obvious as that in patients after SG, but it can make obese patients get the same good weight loss and metabolic improvement as patients after SG. Ghrelin level at the third month after operation is the influencing factor of postoperative weight loss. Further studies should be continued to have significantly sufficient data to permit implementation on a wide scale.

## Abbreviations

SGFD: Sleeve gastrectomy combined with fundoplication
SG: Sleeve gastrectomy
GERD: Gastroesophageal reflux disease
BMI: Body mass index

## Declarations

### Data availability

The data that support the findings of this study are available from the authors but restrictions apply to the availability of these data, which were used under license from the People’s Hospital of Xinjiang Autonomous Region for the current study, and so are not publicly available. Data are, however, available from the authors upon reasonable request and with permission from the Information Center of People’s Hospital of Xinjiang Autonomous Region. If someone wants to request the data from this study, they should contact the corresponding author.
